# Clinical AI in Radiology: Foundations, Trends, Applications, and Emerging Directions

**DOI:** 10.3390/cancers18060942

**Published:** 2026-03-13

**Authors:** Iryna Hartsock, Nikolas Koutsoubis, Sabeen Ahmed, Nathan Parker, Matthew B. Schabath, Cyrillo Araujo, Aliya Qayyum, Cesar Lam, Robert A. Gatenby, Ghulam Rasool

**Affiliations:** 1 Department of Machine Learning, H. Lee Moffitt Cancer Center & Research Institute, Tampa, FL 33612, USA; niko.koutsoubis@moffitt.org (N.K.); sabeen.ahmed@moffitt.org (S.A.); cyrillo.araujo@moffitt.org (C.A.); aliya.qayyum@moffitt.org (A.Q.); cesar.lam@moffitt.org (C.L.); ghulam.rasool@moffitt.org (G.R.); 2 Department of Electrical Engineering, University of South Florida, Tampa, FL 33620, USA; 3 Department of Health Outcomes and Behavior, H. Lee Moffitt Cancer Center & Research Institute, Tampa, FL 33612, USA; nathan.parker@moffitt.org; 4 Department of Cancer Epidemiology, H. Lee Moffitt Cancer Center & Research Institute, Tampa, FL 33612, USA; matthew.schabath@moffitt.org; 5 Department of Diagnostic Imaging & Interventional Radiology, H. Lee Moffitt Cancer Center & Research Institute, Tampa, FL 33612, USA; robert.gatenby@moffitt.org; 6 Department of Oncologic Sciences, University of South Florida, Tampa, FL 33612, USA; 7 Department of Neuro-Oncology, H. Lee Moffitt Cancer Center & Research Institute, Tampa, FL 33612, USA

**Keywords:** radiology, multimodal artificial intelligence, local large language models

## Abstract

Artificial intelligence (AI) is at the vanguard of transforming radiology in several ways, including augmenting diagnoses, improving workflows, and increasing operational efficiency. Several integration challenges, including concerns over privacy, clinical usability, and workflow compatibility, still remain. This review discusses the foundations and current trends of clinical AI in radiology to provide essential context for ongoing developments. To illustrate translational potential, we describe representative applications, including: (1) local deployment of large language models (LLMs) for restructuring and streamlining radiology reports, improving clarity and consistency without relying on external resources; (2) multimodal AI frameworks combining CT images, clinical data, laboratory biomarkers, and LLM-extracted features from clinical notes for early detection of cachexia in pancreatic cancer; (3) privacy-preserving federated learning (FL) infrastructure enabling collaborative AI model development across institutions without sharing raw patient data; and (4) an uncertainty-aware de-identification pipeline for removing Protected Health Information (PHI) from radiology images and clinical reports to support secure data analysis and sharing. We further discuss emerging opportunities for tumor board decision support, clinical trial matching, radiology report quality assurance, and the development of an imaging complexity index. Collectively, these applications highlight the importance of local deployment, multimodal reasoning, privacy preservation, and human-in-the-loop oversight in translating AI models from research to oncology radiology practice.

## Simple Summary

Artificial intelligence (AI) is increasingly being explored in radiology to assist with image interpretation, organize clinical information, and improve workflow efficiency. This review summarizes the foundations of clinical AI in radiology and highlights current trends shaping its development and use in medical imaging. Several representative examples are presented, including locally deployed language models that help structure radiology reports, multimodal AI approaches for early detection of cachexia, federated learning systems that enable collaboration across institutions without sharing patient data, and automated methods for removing identifying information from radiology images and reports. Emerging AI-driven directions are also discussed, including tumor board decision support, clinical trial matching, radiology report quality assurance, and the development of an imaging complexity index. Together, these developments illustrate how AI is gradually being integrated into radiology practice while maintaining clinician oversight and protecting patient privacy.

## 1. Introduction

Radiology plays a central role in oncology, including early detection, diagnosis, staging, treatment monitoring, and post-therapy surveillance. As medical imaging volumes grow and case complexity increases, radiologists face mounting demands for accurate and rapid interpretation, efficient reporting, and multidisciplinary communication [1]. Artificial intelligence (AI) offers a promising solution to support radiologists across these workflows [2]. Large language models (LLMs), such as GPT-4/5, Vicuna, and Rad-Phi2, have been applied to radiology-specific tasks, including structured reporting, automated impression filling, extraction of key diagnostic information from unstructured text, and question answering [3,4,5]. Vision-based models, like U-Net, YOLO, and ResNet, have been increasingly used in radiology image segmentation, detection, and classification tasks [6,7,8]. Advances in multimodal learning and vision language models (VLMs) have introduced new capabilities for combining imaging information with clinical text to enhance diagnostic reasoning and support informed decision-making. Models such as Flamingo CXR (2024) [9], RadVLM (2025) [10], Med3D VLM (2025) [11], RadAlign (2025) [12], and RadZero (2025) [13] enable tasks including automated report generation, visual question answering (VQA), and prognosis prediction, highlighting their potential in oncology and beyond [14,15]. Alongside these systems, several radiology foundation models (FMs) have begun to emerge. RadFM (2023) [16], CURIA (2025) [17], and ONCOPILOT (2025) [18] provide broader, transferable representations that can support a wide range of downstream radiology and oncology tasks, reflecting a shift toward more general purpose radiology AI. As multimodal models continue to grow in scale, standardized preprocessing pipelines are essential for consistent image quality and reproducible model performance. Frameworks such as Pillar-0 provide modular tools for large-scale preprocessing, intensity normalization, and dataset curation, which support the development of both unimodal and multimodal radiology FMs [19].

Despite rapid technical advances, many radiology teams remain cautious about integrating AI tools into daily practice [2]. AI development often occurs in research environments and may not always align with the realities of clinical radiology workflows [20]. Concerns over opaque “black-box” decision-making remain a major barrier to clinical trust [21]. As a result, models may lack the usability, reliability, generalizability, and transparency required at the point-of-care, with diagnostic accuracy issues and the risk of automation bias further limiting adoption. In oncology imaging specifically, where complex, multi-modality assessments and nuanced interpretations are routine, the absence of cancer-focused, radiologist-centered AI tools exacerbates these challenges [22]. Privacy regulations and institutional data governance frameworks may restrict the use of cloud-based solutions, while some models remain proprietary and unsuitable for customization or local deployment. Consequently, AI adoption in radiology remains measured and fragmented, despite radiologists’ interest in tools that may help reduce reporting burden, support diagnostic confidence, and improve workflow efficiency.

Comprehensive cancer centers are placing increasing emphasis on AI solutions that address radiology needs through secure local deployment and clinician-centered design. At institutions such as the H. Lee Moffitt Cancer Center & Research Institute, these considerations have informed the development and evaluation of AI tools tailored to radiology workflows in oncologic practice. Rather than relying on generic commercial platforms, these efforts prioritize adapting state-of-the-art methods to the specific challenges of cancer imaging, including the high prevalence of multi-modality studies, the importance of longitudinal follow-up in oncology care, and the need for clear communication with oncologists. Local deployment ensures that model inference occurs entirely within institutional firewalls, thereby reducing privacy risks and strengthening compliance with regulatory standards. Equally important, clinician input is embedded at every stage of development, from prompt design for report restructuring to validation of multimodal prognostic models, so that tools align with established reading patterns and reporting standards. This emphasis on workflow integration and privacy preservation supports the development of AI systems that are both technically advanced and operationally feasible.

While local deployment and institution-centered development offer advantages for privacy, customization, and workflow integration, they may raise concerns about feasibility for smaller hospitals or institutions without dedicated machine learning teams. In practice, many radiology AI applications can operate on modest infrastructure, such as a workstation with a single GPU or CPU-based inference for smaller models. Hardware demands can be further reduced using lightweight approaches such as quantized or distilled models. Institutions may also adopt AI gradually, beginning with simple tools such as locally deployed inference models or reporting assistants, while components such as model training or federated learning coordination can be shared across institutions.

**Figure 1:**
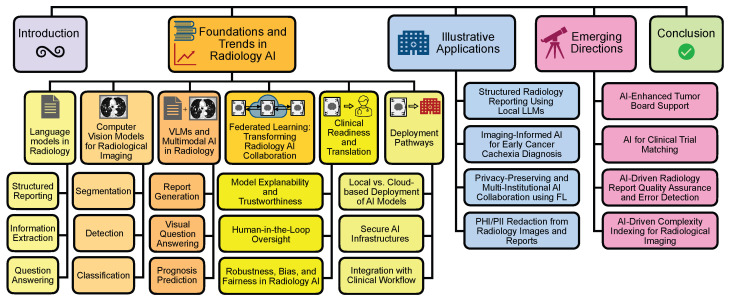
Overview of the paper. The manuscript is structured around three primary components: (1) Foundations and current trends in radiology AI, (2) illustrative applications, and (3) emerging directions in radiology AI.

This manuscript presents a thematic narrative review of key domains shaping clinical AI development and deployment in radiology, drawing on the contemporary literature and selected translational examples to synthesize current trends and implementation considerations. We first review the foundations and current trends in clinical AI for radiology, highlighting advances in language models, computer vision, and multimodal vision–language systems, followed by developments in federated learning, considerations for clinical readiness and translation, and practical deployment pathways (Section 2). We then describe illustrative applications demonstrating practical integration across structured reporting, multimodal cachexia prediction, federated learning, and PHI/PII-redaction workflows (Section 3). Finally, we discuss emerging directions in radiology AI, including tumor board decision support, clinical trial matching, radiology report quality assurance, and imaging complexity index development (Section 4). Our aim is to offer actionable insights for radiology departments seeking to responsibly adopt and integrate AI technologies to enhance diagnostic quality, workflow efficiency, and patient care. The overall organization of the paper is summarized in Figure 1.

## 2. Foundations and Trends in Radiology AI

AI in radiology encompasses a range of models designed to process medical imaging data, clinical text, or multimodal combinations of both [23,24,25]. To support collaboration across institutions while preserving patient privacy, federated learning frameworks enable joint model training without requiring data transfer [26]. Bringing these systems into practice requires careful attention to clinical readiness, encompassing model explainability and trustworthiness, radiologist involvement in the loop, and comprehensive strategies to address bias, fairness, and robustness. Once models reach sufficient maturity, they can be deployed through infrastructures that safeguard data security, minimize technical overhead, and integrate seamlessly into clinical workflows, whether maintained locally or supported externally.

### 2.1. Language Models in Radiology

Text is central to radiology, with radiology reports serving as the primary means of documenting imaging findings, diagnostic impressions, and clinical recommendations. In addition to radiology reports, clinical data is also embedded within the electronic health record (EHR) system, which include clinical notes, pathology reports, lab results, medication lists, and imaging summaries. Recent progress in natural language processing (NLP) has enabled new tools for analyzing and working with textual radiology and diagnosis-related data. Central to this progress is the introduction of transformer-based models, which depart from sequential text processing by analyzing a large set of words simultaneously. Through a self-attention mechanism, transformers capture contextual relationships across entire passages, enabling deeper and more flexible language understanding [27]. Early transformer models such as Bidirectional Encoder Representations from Transformers (BERT) laid the groundwork for modern NLP by introducing bidirectional context processing [28].

Building on this foundation, LLMs have emerged as valuable tools for radiology-specific NLP tasks [29]. These models are trained on vast text corpora and contain billions to hundreds of billions of parameters, enabling them to capture far more complex language patterns and nuances. These models can be fine-tuned on specialized datasets, such as annotated radiology reports, to further improve their performance on targeted clinical tasks [24]. In addition to fine-tuning, prompt-based approaches have become increasingly popular. Rather than modifying the model itself, prompt-based methods guide the model’s behavior through carefully crafted input instructions [30]. This allows general-purpose LLMs to perform domain-specific tasks with minimal setup, offering flexibility and enabling quick adaptation to new tasks. In radiology, these models are increasingly used for structuring reports, extracting clinical information, and question-answering.

#### 2.1.1. Structured Reporting

Structured reporting is one of the examples how NLP can support radiology practice. Studies have shown that structured reports offer greater clarity and completeness compared to traditional free-text reports [31]. Recently, prompting LLMs have been explored as tools for converting unstructured radiology reports into structured formats [32]. Models such as Generative Pre-trained Transformer 4 (GPT-4) and Mixtral have been applied for this task, offering a more flexible and scalable alternative to rigid template-based systems, particularly when combined with domain-specific prompt engineering [3,33].

#### 2.1.2. Information Extraction

Information extraction is another essential NLP task in radiology, aimed at identifying clinical entities such as anatomical structures, imaging findings, and diagnostic impressions, as well as markers of uncertainty or temporal information. Extracting these elements accurately is crucial for downstream applications like clinical decision support, research analytics, and integration with structured EHRs. Traditionally, transformer-based models such as BERT have been widely used for this task, often with domain-specific fine-tuning to enhance performance on radiology texts [34]. More recently, prompt-based approaches using open-source LLMs like Vicuna have shown strong results in extracting structured information from unstructured radiology reports, leveraging well-crafted prompts to reduce the need for additional training [4].

#### 2.1.3. Question Answering (QA)

QA is an emerging area in radiology where language models are used to respond to natural language queries. This is particularly valuable in clinical settings where rapid access to specific findings is needed. For instance, a model might answer questions like “Is there any evidence of pleural effusion?” or “Has this thyroid cancer progressed?” [35]. Besides radiology reports, questions may draw on broader content from EHRs. Clinical LLM such as GatorTron has demonstrated strong performance in clinical QA tasks involving EHRs, although it was not specifically trained on radiology data [36]. Beyond clinical use, LLMs are increasingly explored for patient-facing applications. When integrated into patient portals, they can help interpret radiology results, explain terminology, and clarify follow-up instructions [37]. LLM-based chatbots, including ChatGPT, Copilot (formerly Bing), and Gemini, have demonstrated high accuracy in responding to patient medical questions, supporting their role in patient education and communication [37,38,39]. However, unmediated patient-facing LLM-based QA raises concerns related to misinterpretation of indeterminate imaging findings and loss of clinical context, underscoring the need for clinician-supervised deployment [40].

### 2.2. Computer Vision Models for Radiological Imaging

A substantial portion of clinical AI in radiology focuses on the interpretation of medical images, such as computed tomography (CT), magnetic resonance imaging (MRI), positron emission tomography-computed tomography (PET-CT), and radiographic “X-ray” scans. Image-based models used in this context generally fall into three categories: convolutional neural networks (CNNs), vision transformers (ViTs), and hybrid architectures that combine the two [41,42,43].

CNNs are the most widely used in radiological visual recognition tasks. These models work by scanning images with small filters to detect local patterns such as edges, shapes, and textures [44]. ViTs are a newer class of models that divide an image into patches, analogous to tokens in text, and process these patches in parallel while learning the relationships between them [45]. This allows ViTs to capture fine-grained spatial detail along with broader contextual cues, making them well-suited for radiology tasks where subtle, distributed patterns are often diagnostically important [46,47]. Unlike CNNs, which process data hierarchically, transformers evaluate spatial relationships more holistically. However, ViTs typically require larger datasets and greater computational resources, which has limited their widespread adoption in clinical workflows thus far [45,47]. Recent approaches have also begun to integrate CNNs with transformers, leveraging the strengths of both: the local feature extraction of CNNs and the global contextual understanding of transformers. In radiology, image-based models are often used for segmentation, detection, and classification tasks [48].

#### 2.2.1. Segmentation

Segmentation involves outlining regions of interest including organs, nodules, tumors, or edema. When done by hand, this process can be time-consuming and may vary between doctors. AI-based methods help by making segmentation faster, more consistent, and less dependent on individual judgment [49]. One of the most widely used architectures for medical image segmentation is U-Net, a CNN-based model with an encoder–decoder structure and skip connections to preserve spatial resolution [6]. Originally designed for 2D image segmentation, U-Net processes individual slices and is often applied to X-rays, ultrasound, or single CT/MRI slices [6,50,51,52]. For volumetric data, 3D U-Net extends this architecture to entire image volumes, capturing spatial context across slices, especially useful for CT and MRI [41]. Numerous other variants of U-Net have been developed to improve performance in radiological applications [53,54]. Despite their success, CNN-based models can struggle with modeling long-range spatial dependencies in high-resolution images.

ViTs have been explored as a solution to this limitation [42,55]. Moreover, hybrid architectures integrate ViTs with CNNs to benefit from both local and global information [46]. A common approach is to incorporate ViTs into different segments of the U-Net architecture. For instance, ViTs can be integrated into the encoder to enhance feature representation [47], into the decoder to improve the reconstruction of segmentation maps from encoded features [56], or into the skip connections to enable richer fusion of local and global features across network levels [57].

#### 2.2.2. Detection

Object detection refers to identifying the presence and location of abnormalities, such as lung nodules, brain lesions, or bone fractures. This typically involves highlighting suspicious regions with bounding boxes and classifying their type. CNN-based object detection models are widely used in medical image detection, and are typically classified as two-stage or single-stage models [58]. Two-stage models first propose regions of interest and then classify them [59]. Single-stage models perform detection and classification in one pass, offering greater speed and efficiency [7,60]. In addition to 2D CNNs, 3D CNNs have been developed to handle volumetric medical data more effectively [61]. More recently, ViTs have been explored for medical image detection, primarily for 2D tasks, due to their ability to capture long-range dependencies. However, their reliance on large datasets and high computational resources has limited their standalone use. Instead, ViTs are often combined with CNNs in hybrid architectures, where they can be integrated into the backbone, neck, or head of the network to enhance feature representation and improve detection performance [62].

#### 2.2.3. Classification

Image classification is the task of assigning diagnostic labels to images or localized regions, such as distinguishing between benign and malignant findings or identifying tumor subtypes. CNNs (e.g., ResNet, DenseNet) are the backbone of most classification systems due to their proven ability to detect patterns in both 2D and 3D imaging data [8,63]. ViTs have also been used for classification tasks, showing stronger performance than CNNs [64,65]. Combining ViTs with CNNs has become increasingly popular as well [66]. Some models, such as TransMed, extend image-based classification by incorporating multiple MRI modalities, specifically T1-weighted and T2-weighted scans, using a hybrid CNN–Transformer architecture [43]. Transfer learning is widely used in this domain, particularly with CNNs, where models pre-trained on large natural image datasets like ImageNet are fine-tuned on smaller medical datasets [67]. This approach significantly improves performance, especially when labeled data is limited. More recently, transfer learning has also been applied to ViTs, though it remains less common due to the larger data and compute requirements for effective pretraining [64]. Radiology classification tasks often involve multi-label outputs since a single scan can reveal multiple co-existing pathologies which requires models that can handle overlapping labels [63].

### 2.3. Vision-Language Models (VLMs) and Multimodal AI in Radiology

Recent advances in AI have led to the development of VLMs that process both visual and textual data [14]. In radiology, this often involves combining visual information from imaging with associated clinical text such as pathology reports, patient history, or lab results. By jointly reasoning over both text and image data, these models provide richer contextual understanding than either modality alone [25].

VLMs are typically organized into single-stream and dual-stream architecture designs [14]. Single-stream models process both image and text data jointly within a unified transformer architecture, allowing for deep interaction between modalities from the early layers. These models often incorporate a vision encoder, such as a CNN or ViT, followed by a projection module that maps the visual features into the same feature (or embedding) space as text tokens. The projected visual features are then combined with tokenized text inputs and jointly fed into an LLM [68,69]. Dual-stream models use separate encoders for image and text inputs without sharing parameters. For example, images may be encoded using a CNN or ViT, while text is processed using an LLM. The modality-specific representations are then aligned at later stages, often using attention-based fusion layers [70]. In models that generate free-text outputs, such as radiology reports or answers to clinical questions, a decoder module is typically introduced in the end [68,70]. In radiology, VLMs are commonly applied to tasks such as report generation, VQA, and prognosis prediction.

#### 2.3.1. Report Generation

Radiology report generation involves identifying key findings, assessing their clinical significance, and clearly communicating the results of imaging studies, such as X-rays, CT scans, or MRIs, to referring physicians. This task is time-consuming and subject to variability in style and completeness. Leveraging VLMs can streamline this workflow by automatically generating structured reports directly from medical images [14]. Most existing VLMs focus on chest X-rays, primarily due to the availability of large paired image–text datasets such as MIMIC-CXR [71]. Recent models including XrayGPT [72], RaDialog [69], RadFM [16], Flamingo-CXR [9], and RadAlign [12] have been fine-tuned on MIMIC-CXR to capture radiology-specific language and image features.

In contrast, there has been comparatively less work on generating radiology reports from 3D radiology images, such as CT and MRI. Med3D-VLM [11] and Med-Gemini-3D [15] integrate 3D medical image encoders to support volumetric modalities, while RadAlign [12] and RadVLM can handle multiple imaging types, including X-ray, CT, and MRI. In report generation, models often utilize task-specific prompts to guide their output toward a desired structure, thereby improving clarity and completeness. Performance can be further enhanced by leveraging CheXpert [73] labels. For example, through a double-feature transformer that integrates a pretrained CNN encoder, such as ImageNet, with a chest radiograph-specific CNN encoder trained on CheXpert labels [74]. More recently, retrieval-augmented generation (RAG) has been incorporated to retrieve relevant, clinically validated report segments from large datasets, resulting in reduced hallucinations and improved factual grounding [12,75].

#### 2.3.2. Visual Question Answering (VQA)

In radiology, VQA requires the model to interpret an image together with a question to produce an accurate and relevant answer. Queries can range from identifying abnormalities or medical conditions to specifying the imaging modality, anatomical region, or presence of certain structures. They are generally open-ended, allowing detailed free-text responses, or closed-ended, with predefined answers such as “yes” or “no,” multiple-choice options, or numeric values. VQA can be approached as a classification task, selecting from a fixed set of answers, or as a generation task, producing responses in free form [14]. Models are frequently fine-tuned on datasets such as VQA-RAD [76] and SLAKE [77], which include X-rays, CTs, and MRIs. Recent approaches also integrate RAG to improve factual accuracy and reduce hallucinations by grounding answers in clinically verified text corpora [78].

Conversational VQA extends standard VQA by enabling multi-turn, context-aware interactions in which follow-up questions can refer back to prior questions or answers. This allows radiologists or clinicians to conduct a dialogue with the AI system; for example, first asking whether a lesion is present, then requesting its size or location without having to restate the full context. Such systems require maintaining conversational memory and reasoning across turns, often integrating dialogue management components on top of standard VQA pipelines. The most recent VLMs that support this task include RaDialog [69] and RadVLM [10].

#### 2.3.3. Prognosis Prediction

Prognosis prediction involves estimating a patient’s future clinical outcome or the likely course of a disease, using imaging, often in combination with other clinical or demographic information. Typical objectives include forecasting survival duration, assessing the probability of disease progression, or determining the risk of recurrence. One recent multimodal approach for pulmonary embolism survival estimation integrates CT pulmonary angiography (CTPA) imaging with automatically generated report segments from a VLM, abnormality classification outputs, and Pulmonary Embolism Severity Index (PESI) scores, with the combined data analyzed using a Cox regression framework [79]. Another example is a three-dimensional FM for abdominal CT that jointly processes volumetric CT data, corresponding radiology reports, and structured diagnosis codes from EHRs. This system supports a range of downstream applications, including the prediction of chronic disease occurrence over a five-year period [80].

### 2.4. Federated Learning: Transforming Radiology AI Collaboration

Training accurate and generalizable radiology models requires access to very large datasets. Most individual institutions have limited patient volumes and do not capture the full diversity of cancer types, stages, and clinical presentations needed for robust oncology research [81]. Privacy regulations such as the Health Insurance Portability and Accountability Act (HIPAA) in the United States and the General Data Protection Regulation (GDPR) in Europe place strict conditions on the transfer of PHI [82,83]. These regulations do not prohibit collaboration, but they require strong safeguards and formal approvals for any movement of patient data. Because these requirements can be complex and time consuming to satisfy, many institutions are unable or unwilling to send patient data to a shared central location, which makes centralized research datasets difficult to assemble at large scale.

**Figure 2:**
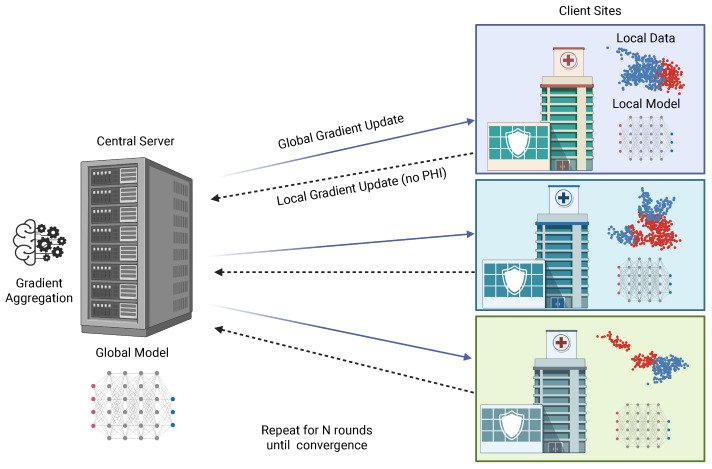
Overview of the FL process. Each institution trains a local model on its own data and sends only updated model parameters to a coordinating server, which produces a global model by aggregating the local updates.

FL offers a practical way to enable collaboration under these constraints [26]. In the FL framework, each institution keeps its data within its own secure environment and trains a local version of the model on its own dataset. After one round of training, the institution sends only the updated model parameters to a coordinating server. The server combines these updates to form a global model and then returns the global parameters to all participating sites. This process repeats for many rounds and is shown in Figure 2. The approach was first formalized in the Federated Averaging (FedAvg) method, in which the global model is produced by computing a weighted average of the local updates [84].

FL has been applied in research across many radiology tasks [26,85]. The Federated Tumor Segmentation effort, which developed from the BraTS initiative, is the largest medical federated project, with participating sites on six continents [86]. FL also enables multimodal development, allowing imaging, clinical notes, laboratory values, and electronic health record information to be used collectively while all data remain within the institution where they were generated [87].

Although FL avoids direct data sharing, it still faces privacy and security challenges [88]. Local model updates can, in some cases, leak information about the underlying data, and studies have shown that sensitive details can sometimes be inferred through attacks that attempt to reconstruct patient information from gradients [88,89] and variety of other attacks [90]. Modern FL frameworks include several safeguards to reduce these risks. Secure aggregation protocols prevent reconstruction of local updates during transmission [91]. Differential privacy adds carefully controlled noise to the model parameters to reduce the chance of patient re-identification [92,93,94]. Homomorphic encryption allows calculations to be performed on encrypted numeric values, maintaining confidentiality throughout the process [95,96]. While these techniques improve privacy protection, they may introduce computational overhead or modest reductions in model performance depending on the privacy parameters used. For this reason, both model performance and privacy risk must be carefully evaluated when deploying FL systems in clinical environments. Taken together, these features make FL a promising path toward large scale collaborative radiology research that preserves both data utility and patient privacy.

### 2.5. Clinical Readiness and Translation

Despite rapid advancements in the capabilities of AI models, successful integration into radiology clinical practice requires ensuring clinical readiness and effectively translating these capabilities into practical applications.

#### 2.5.1. Model Explainability and Trustworthiness

For radiology AI models to be clinically adopted, they must produce outputs that radiologists can interpret and verify. Black-box predictions undermine trust, particularly in high-stakes diagnostic contexts such as oncology. A range of explainability techniques can be applied, depending on the model type, whether it is language-based, vision-based, or a multimodal VLM.

For language-based radiology AI, attention weight visualization is a common approach for identifying which tokens a model attends to during prediction. However, its validity is not fully established, as attention does not always align with the truly influential features [97]. One alternative is TokenSHAP, an adaptation of SHapley Additive exPlanations (SHAP) [98] tailored to sequential data [99]. TokenSHAP assigns each token an importance value based on cooperative game theory, while preserving the dependencies between words, and offers a more reliable measure of token influence than raw attention scores alone. Building on attribution-based insights, chain-of-thought reasoning offers a complementary perspective by revealing the model’s intermediate reasoning steps, either extracted from hidden states or generated explicitly [100]. Recent approaches enhance this by adding an explicit reasoning component, where an LLM generates not only its final output but also a human-readable explanation grounded in evidence from the input [101]. This reasoning trace can then be audited for factual accuracy, increasing trust in AI-assisted decision-making.

In computer vision models, guided backpropagation examines how a model’s output changes when parts of the input image are perturbed, helping to identify which pixels most influence the prediction [102]. Techniques based on the class activation mapping (CAM) [103], such as Grad-CAM [104] and Score-CAM [105], localize the image regions that contribute most to a model’s decision, providing a visual heatmap of the most important features. Concept Activation Vectors (CAVs) measure how strongly a clinically relevant concept (e.g., a lung nodule) influences a model’s predictions across many cases [106]. Unlike previous methods, which explain a single prediction, CAVs enable the global testing of concepts, making them valuable for detecting hidden biases and verifying that the model bases its decisions on medically meaningful features rather than spurious patterns. In radiology, for example, Grad-CAM has been used to highlight critical areas in brain MRIs [107], Score-CAM has been applied to mammography images [108], and CAVs have been employed in chest X-ray analysis [109].

In the context of VLMs for radiology, newer adaptations extend these ideas to align image regions with generated text. VL-InterpreT is an interactive visualization system designed for multimodal transformers [110]. It enables the examination of both intra- and cross-modal attention patterns and tracks how hidden representations evolve across layers for both image and text tokens. MM-SHAP [111] adapts the SHAP framework to multi-modal models, assigning importance scores to both text tokens and image patches. However, its reliance on patch-level granularity, rather than semantically meaningful objects, limits the clinical interpretability of its visual outputs. PixelSHAP [112] builds on this approach for text-generative VLMs by attributing importance to structured visual entities (e.g., anatomical regions), producing explanations that are more aligned with human visual reasoning.

#### 2.5.2. Human-in-the-Loop Oversight

Human-in-the-loop (HITL) oversight refers to the continued involvement of qualified professionals in the operation of an AI system, ensuring that model outputs are reviewed and corrected when necessary before they influence patient care [113]. This is particularly important in radiology, where diagnostic decisions carry high clinical stakes and imaging findings vary widely across patient populations. HITL ensures that responsibility for the final interpretation remains with the radiologist, positioning AI as an assistive rather than an autonomous agent.

HITL engagement occurs across multiple stages of the AI life cycle. During development, radiologists may annotate datasets, validate algorithmic outputs, and provide structured feedback that guides the refinement of the model. Active learning approaches exemplify this stage: by algorithmically identifying high-yield or uncertain cases for expert labeling, these methods reduce annotation burden while ensuring that physician expertise is directed where it is most needed [114]. Reinforcement learning with human feedback (RLHF) has also been proposed as a complementary strategy, where expert evaluations are integrated into training to align models with clinically meaningful outcomes [115].

In deployment, HITL most often takes the form of radiologists reviewing and editing AI-generated outputs before they are entered into the official patient record. This preserves diagnostic authority while enabling workflow gains. A recent study showed that radiologists using AI-assisted draft reports achieved a reduction in average reporting time from 573 to 435 s without an increase in clinically significant errors [116]. Gaze tracking-based oversight has also been proposed, where eye movement data highlight regions that may have been overlooked by either the AI or the radiologist, thereby reducing perceptual misses [117]. In some deployments, clinician adjustments to AI outputs are recorded and later reviewed in local governance meetings, providing a structured feedback mechanism for oversight [118]. Other work has demonstrated frameworks where AI results are delivered through DICOM structured reporting, allowing radiologists to directly validate or reject outputs in their reading environment and ensuring a transparent feedback loop between the system and its users [119].

Despite these safeguards, HITL must also address automation bias, a cognitive risk that arises when clinicians interact with algorithmic outputs. Automation bias refers to the tendency to rely too heavily on AI suggestions and insufficiently question incorrect results. Studies have shown that AI suggestions can influence interpretation, including greater acceptance of false-positive findings and changes in follow-up recommendations when the AI output is incorrect [120]. These observations indicate that the presence of human review alone does not eliminate risk; the clinical workflow itself must support independent judgment.

Mitigating automation bias therefore requires safeguards embedded within routine practice. In some settings, delaying exposure to AI outputs until after an initial review may reduce early anchoring to algorithmic suggestions. Where available, presenting confidence levels or uncertainty indicators alongside AI findings can also help communicate that outputs may vary in reliability. Active confirmation of high-risk findings prior to report finalization, along with periodic review of AI–radiologist disagreement cases within quality governance processes, may help identify systematic failure patterns. Education that familiarizes clinicians with known model limitations and common error modes further supports appropriate clinical scrutiny.

#### 2.5.3. Robustness, Bias, and Fairness in Radiology AI

Many radiology AI models perform well on retrospective or single-institution datasets but often lose accuracy when applied across broader clinical populations. In a review of 83 published radiology AI studies, almost half of the models that underwent external validation showed at least a moderate drop in accuracy, and nearly one-quarter experienced a major decline compared with their internal test results [121]. For example, the ICOVAI system for COVID-19 CT and several mammography models both showed strong internal performance but declined when tested on external cohorts [122,123]. Another model trained for pneumonia detection relied partly on hospital-specific image characteristics, leading to reduced diagnostic reliability at external sites [124]. These findings illustrate the need for systematic testing across diverse sites before clinical adoption.

Performance differences are not only institutional but also demographic. For instance, one study showed that models trained on male-dominant datasets demonstrated reduced accuracy in female patients, creating systematic bias in chest pathology detection [125]. Another work has demonstrated that race can be inferred from medical images even though it is not visible to human readers, raising the concern that such hidden signals may influence predictions in unintended ways [126].

Several approaches are being developed to address these challenges. FL allows collaborative training of models across institutions without sharing raw data (e.g., DICOM images), and it can achieve performance that is comparable, and even superior, to centralized models while maintaining privacy and institutional control [127,128]. The federated models, owing to the way they are trained, may result in more generalizable models addressing the challenges of models trained using a single side data. Domain adaptation seeks to harmonize imaging data from different scanners or acquisition protocols so that models trained in one environment remain reliable in another. For example, attention mechanisms can guide models toward features that remain consistent across sites, while disentangled networks can harmonize scans to appear more comparable without retraining [129,130]. Data augmentation also plays a role in improving generalization and addressing data imbalance. Diffusion models generate high fidelity synthetic medical images by learning to reverse a gradual noising process, allowing realistic creation of CT and MRI examples that expand limited datasets and support more robust model performance across varied clinical settings [131]. To reduce demographic bias, techniques such as REPAIR reweight training samples to encourage learning of features that generalize beyond overrepresented groups [132]. Adversarial debiasing takes a different approach, using an auxiliary network to detect sensitive attributes and penalizing the predictor when those attributes are encoded, producing outputs that are less dependent on demographic factors [133].

### 2.6. Deployment Pathways

Effective radiology- AI deployment depends on where models run, how securely and reliably they are operated, and how seamlessly they fit in the clinical workflow.

#### 2.6.1. Local vs. Cloud-Based Deployment of AI Models

Hospitals that want to use AI in radiology face an important choice: deploy the AI models on local computation hardware or connect to the provider’s remote servers (i.e., cloud-based deployment). Local deployment means the model runs on servers that are physically available inside the hospital’s network. For example, platforms such as Ollama and vLLM allow LLMs/VLMs to be downloaded and run locally [33]. Local systems may have lower latency because they avoid delays introduced from sending data over the internet. However, local deployment requires institutions to invest in computer equipment such as graphics processing units (GPUs) and to maintain software updates themselves.

Cloud-based services work differently. Data are sent over secure internet connections to a vendor’s servers, where the AI model are deployed. The results are then sent back to the institution. This arrangement reduces the technical burden for the institutions and ensures they are always using the latest model version. Well-known examples include Viz.ai, RapidAI, and Aidoc, which provide FDA-cleared cloud-based radiology AI applications for stroke triage, pulmonary embolism detection, and other urgent findings. General-purpose platforms such as Amazon Web Services (AWS) also provide the infrastructure to host radiology AI at scale, and many vendors build their services on top of these environments. The trade-off is that data leave the institution, so hospitals must rely on strong encryption, legal agreements, and regulatory compliance to protect PHI. In most cases, these services are accessed through application programming interfaces (APIs), which act as standardized gateways for sending data to the vendor and receiving model outputs in return. APIs make integration with existing systems easier but also create another layer of dependency on the vendor’s infrastructure and uptime.

To simplify both approaches, many developers now deliver their software in containers (e.g., Docker). A container packages the AI model together with all the source code and dependence libraries it needs to run, so the same container can be installed locally behind the firewall or deployed on cloud servers by the vendor. In radiology AI, a leading example is the MONAI Deploy Toolkit, which is designed to streamline the packaging, validation, and integration of AI applications into clinical imaging environments [20]. Using this framework, AI models can be packaged into containers that connect directly with institutional systems, including the Picture Archiving and Communication System (PACS) and Radiology Information System (RIS). This setup allows imaging studies to be processed securely and re-integrated into clinical workflows within the institutional firewall.

#### 2.6.2. Secure AI Infrastructures

The deployment of AI in radiology requires more than deciding between local or cloud-based hosting. It also depends on secure infrastructures that can protect imaging data, maintain reliable performance, and integrate safely into clinical workflows. Because radiology data contain sensitive personal and diagnostic information, breaches or failures can have significant clinical and legal consequences.

A core requirement is cybersecurity. Imaging networks have been frequent targets of ransomware, and vulnerabilities in PACS and hospital IT systems have been exploited in the past [134]. Effective safeguards include proactive vulnerability assessments, strong authentication, encryption of imaging data in transit and at rest, and formal incident response protocols [135]. These protections are especially critical when AI tools integrate with PACS, RIS, or EHRs, where unauthorized access could expose both imaging data and clinical metadata.

Equally important is lifecycle monitoring of deployed models. Unlike static software, radiology AI can lose accuracy over time as scanners are upgraded, imaging protocols evolve, or patient populations shift. Without oversight, this “model drift” can reduce clinical reliability. Continuous monitoring frameworks have been proposed in radiology to track performance in real-world use, flag model decline, and trigger model recalibration or retraining when needed. Some authors describe this as the move toward continuous learning AI, which brings both opportunities for adaptation and new requirements for governance and validation [136].

Finally, institutional governance plays a central role. Many institutions are beginning to establish AI oversight committees or integrate AI risk management into existing quality assurance programs [118]. These groups review proposals for new AI deployments, set guidelines for validation, and monitor safety once systems are in use. Such governance ensures that radiology AI does not function as a “black box” add-on but as a monitored tool within the broader framework of patient safety and institutional accountability.

#### 2.6.3. Integration with Clinical Workflow

Beyond the decision to host applications locally or in the cloud, and aside from the technical safeguards that protect medical data, the long-term value of radiology software depends on whether it fits smoothly into day-to-day workflow. Radiologists are far more likely to use decision-support tools when results appear directly in the software programs they already depend on, such as PACS viewers, structured reporting systems, or electronic templates. Research has shown that AI applications must be presented with minimal extra steps and implemented in a way that does not disrupt existing reporting routines; otherwise, they are unlikely to be embraced in daily practice [137].

One way hospitals are addressing this problem is by adopting vendor-neutral platforms. For example, the ROCKET platform routes studies to selected algorithms and returns the findings into PACS using DICOM Structured Reporting. This setup allows radiologists to review results in the same environment where they interpret images and also provides mechanisms to accept, reject, or request revisions, ensuring that AI remains embedded within the reporting workflow [119].

Institutions are also beginning to adopt vendor-neutral AI (VNAI) platforms to simplify integration at scale. These platforms serve as an intermediary between institutional imaging infrastructure and a range of AI applications. They handle data routing, standardize interfaces, and return outputs directly into PACS or reporting systems. By consolidating connections, VNAI platforms reduce technical overhead and give institutions the flexibility to introduce or retire applications without being locked to a single supplier. This architecture makes it possible to support multiple clinical use cases while keeping results embedded in the radiologist’s familiar workflow [138].

## 3. Illustrative Applications

The following applications are presented as illustrative case studies demonstrating how AI systems can be developed and deployed within real-world clinical environments. While these examples reflect implementations within a specific institutional context, they highlight transferable design principles for clinical AI development and deployment. For each use case we discuss practical considerations such as compute requirements, data scale, evaluation strategies, and deployment constraints to support adaptation across institutions with different infrastructure and regulatory environments.

### 3.1. Structured Radiology Reporting Using Local LLMs

Radiology reporting plays an essential role in cancer care by guiding diagnostic interpretation, staging, and treatment planning. However, radiology reports can often be lengthy, formatted in various ways, and variable between individual radiologists [139]. This lack of standardization poses challenges for referring oncologists, who may be interested in quickly extracting key diagnostic information to inform patient management. Furthermore, equally important is the fact that these radiology reports will serve as a data point for future AI model training. Consistently structured reports therefore become an important asset for future AI development. We identified the need for a scalable and privacy-preserving solution to improve the clarity, conciseness, and structural consistency of radiology reports, particularly in the context of high-volume oncology imaging.

To address this need, we developed and implemented an LLM-based radiology report processing pipeline aimed at restructuring and streamlining radiology reports [33]. A central design priority was safeguarding patient privacy by ensuring that all model inference occurred locally, behind our institutional firewall. This local deployment strategy allowed us to avoid reliance on external cloud-based APIs, thus eliminating the risks associated with transmitting PHI outside institutional control.

Our technical workflow involved evaluating several open-weight state-of-the-art LLMs, such as Mixtral [140], Mistral [141], and Llama 3 [142]. All models were deployed on institution-owned workstations equipped with standard hardware configurations, including RTX 3060 GPUs with 12 GB of VRAM. The Ollama framework facilitated efficient local inference, and LangChain was used to implement flexible prompt engineering workflows tailored to radiology-specific tasks.

A key component of our development process involved optimizing prompt strategies to balance two primary goals: reducing report verbosity and ensuring its structural consistency. In collaboration with board-certified body radiologists, we designed and tested five distinct prompting strategies. Among these, the two-step *Conciseness then Structure (C >> S)* strategy consistently produced the most reliable results. This approach first condensed the original report to eliminate redundancy and unnecessary phrases, followed by a second step that reorganized the content into a standardized, organ-based template. We also incorporated an automated output validation step using an *OutputFixingParser*, which prompted the LLM to correct its formatting if initial outputs did not follow the desired structure.

In a retrospective quality improvement study, we applied the LLM pipeline to 814 de-identified radiology reports covering CT scans of the chest, abdomen, and pelvis from cancer patients. These reports were authored by seven board-certified radiologists at our institution. The results demonstrated a reduction in report length, with an average word count decrease exceeding 53% across all reports. The LLM-processed reports also exhibited greater structural uniformity, potentially improving the accessibility of key findings for referring oncologists. Preliminary feedback from radiologists underscored the perceived benefits of the tool in enhancing report readability and communication with clinical teams. However, this work was not designed as a formal clinical validation study; therefore, we did not perform blinded reader assessments, structured semantic error analysis, or quantitative evaluation of clinical decision-making impact.

Building on these initial findings, we have recently expanded our LLM-based radiology report pipeline to incorporate reasoning-focused LLM capabilities. A recognized limitation of current LLM outputs is the absence of transparent, interpretable reasoning behind the generated content. To address this, we now employ reasoning-enabled models, including DeepSeek [101] and QwQ [143]. These LLMs extract and present intermediate reasoning steps during report transformation to the user for transparency and validation. These reasoning traces include justifications for why certain findings were categorized under specific organ systems. By exposing this internal decision-making process, we aim to establish a more interactive AI–human feedback loop. While reducing redundancy remains beneficial, in subsequent iterations the emphasis shifted away from conciseness after recognizing that excessive compression may risk loss of important clinical information, particularly in complex oncology imaging. As a result, preserving critical clinical detail and minimizing the risk of unintended information loss became central design priorities. Currently, three board-certified body radiologists are conducting a review of both the final structured reports and the underlying reasoning pathways, to provide feedback for informing further model refinement and prompt optimization.

Through this iterative approach, we are advancing toward a more explainable and collaborative paradigm of AI deployment in radiology. The lessons learned from this application, particularly around local deployment feasibility, prompt strategy design, and clinician–AI interaction, are informing broader efforts toward responsible AI development in radiology reporting. This experience highlights the feasibility of developing and refining locally deployed LLM-based tools for radiology reporting workflows in oncology settings.

For institutions interested in adopting a similar approach, the pipeline relies on prompt engineering with open-weight LLMs in inference mode and does not require model training or fine-tuning. Adoption therefore involves defining a report structure appropriate for the specific CT study type, as different CT examinations (e.g., chest, abdomen/pelvis, angiography) require distinct organizational frameworks. Institutions may begin with the structure presented in [33] for chest, abdomen, and pelvis CT and refine or redesign templates and prompts to accommodate other study types. Smaller or quantized models can run on a single GPU workstation (e.g., 12 GB VRAM), and in some cases CPU-based inference may be feasible depending on model size and performance expectations. Because no model training is required, large-scale datasets are not necessary; instead, institutions should validate the pipeline on a representative sample of reports for each CT type to assess structural consistency and information preservation. Before routine use, local testing with radiologists and institutional IT teams is recommended to confirm that outputs align with existing reporting practices and data governance requirements, with ongoing periodic review after deployment.

### 3.2. Imaging-Informed AI for Early Cancer Cachexia Diagnosis

Cancer cachexia, a multifactorial metabolic syndrome characterized by substantial weight loss, skeletal muscle wasting, and systemic inflammation, remains one of the underdiagnosed oncology conditions despite its profound impact on patient survival and quality of life. Up to 80% of cancer patients are affected by cachexia with high prevalence in certain cancers, such as gastroesophageal, pancreatic, colorectal, lung, and hematological [144,145,146]. Existing diagnostic mechanisms are often limited by manual processes, fixed thresholds, and poor integration into clinical workflows [147,148,149]. To address this gap, we have developed clinically deployable AI tools to bridge this gap, focusing on reliability, interpretability, and workflow compatibility to bring imaging intelligence directly into patient care.

Skeletal Muscle Assessment–Automated and Reliable Tool based on AI (SMAART-AI) introduces an uncertainty-aware imaging data processing framework for automated skeletal muscle segmentation from routine CT scans. Rather than optimizing for accuracy alone, SMAART-AI prioritizes reliability and trustworthiness, a critical step in clinical deployment [150,151]. Unlike existing commercial segmentation tools that may silently degrade under input data domain shifts (e.g., differences in scanner type, protocol, image quality, population demographics, or events like a pandemic), SMAART-AI quantifies prediction uncertainty and flags potentially unreliable outputs, enabling HITL validation. Training strategies and reliability-based design make the tool robust in heterogeneous cancer cohorts. SMAART-AI also supports longitudinal tracking of skeletal muscle area (SMA) and skeletal muscle index (SMI), providing insight into changes throughout the treatment journey. The imaging-derived metrics are further integrated with structured and unstructured patient data for downstream tasks such as cachexia risk and survival prediction.

SMAART-AI implements a standardized CT preprocessing and segmentation workflow in which axial CT series are identified, the L3 vertebral level is localized, and a 2D nnU-Net-based model segments skeletal muscle on the selected L3 slice to compute SMA and height-normalized SMI. To improve robustness and quantify epistemic uncertainty, segmentation uses a deep ensemble trained with 5-fold cross-validation across two independently trained architectures. Specifically, for each fold, both architectures are trained from random initialization, yielding a 10-model ensemble (5 folds × 2 architectures), which improves prediction stability under limited labeled training data and heterogeneous imaging conditions. The models were trained on gastroesophageal and pancreatic L3 CT images and evaluated on held-out test images not used during training. At inference, the final muscle mask is obtained by pixel-wise averaging across all ensemble members, and uncertainty is computed from the variance across ensemble outputs, enabling reliability triage and expert review for flagged cases. The pipeline was evaluated across four heterogeneous cohorts, including gastroesophageal, colorectal, pancreatic, and ovarian cancer, and benchmarked against available reference standards, primarily expert manual SliceOmatic annotations and, where available, clinical/commercial tool measurements, ensuring that downstream multimodal analyses use quality-controlled SMA/SMI features under real-world imaging variability.

**Figure 3:**
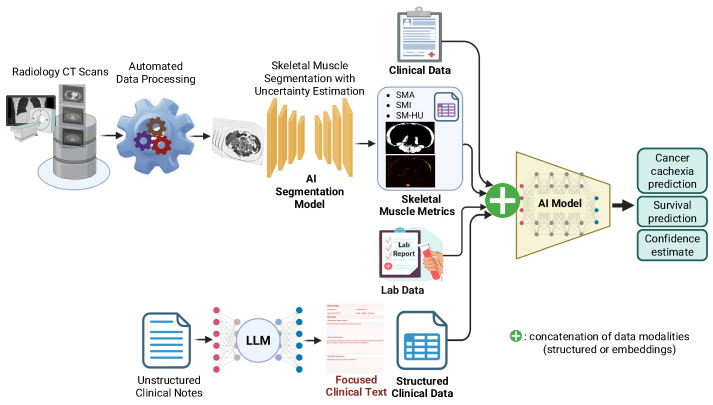
Multimodal AI workflow for cachexia prediction. The framework integrates imaging-derived skeletal muscle metrics generated by SMAART-AI with structured clinical variables, laboratory biomarkers, and cachexia-related symptoms extracted from unstructured physician notes using locally deployed LLMs. Each data modality is processed through dedicated feature extraction modules and fused into a unified multimodal representation for cachexia classification and survival prediction.

Building on SMAART-AI, we developed a “Multimodal AI Biomarker” framework that extends radiologic insight into a broader, data-driven pipeline for early cachexia diagnosis (see Figure 3). The Multimodal AI Biomarker fuses CT-derived skeletal muscle metrics with structured clinical variables (e.g., age, sex, race, ethnicity, weight, height, BMI, and stage of disease), blood-based laboratory biomarkers (e.g., serum albumin, neutrophil, lymphocyte count, blood, urea, nitrogen, creatinine, and derived ratios such as neutrophil-to-lymphocyte and blood-urea-to-creatinine), and cachexia-related symptoms extracted from unstructured clinical notes using locally deployed LLMs. The framework adapts dynamically to patient-specific factors and missing data modalities, ensuring compatibility with real-world clinical data and readiness for potential clinical deployment.

A retrospective dataset of 236 patients with pancreatic cancer from the Florida Pancreas Collaborative cohort was used to build the multi-modal model. To reduce overfitting given the modest cohort size and high-dimensional multimodal embeddings, we used a strict evaluation protocol with an independent held-out test set that was not used during model selection. Cross-validation and hyperparameter selection were performed only within the training split (Cohort I: 10-fold CV on *n* = 210; Cohort II: 7-fold CV on *n* = 105), and final performance was reported on the held-out test set (*n* = 26 for each cohort). Hyperparameter optimization was restricted to training folds only. The fusion MLP includes a learned bottleneck (first hidden layer) that compresses the concatenated embeddings and functions as a data-driven dimensionality reduction step. Regularization was applied via dropout and L2 weight decay, and early stopping was used based on validation loss. Final patient-level predictions were obtained by averaging across folds and across five MLP configurations to reduce variance. Future work will evaluate the approach on external multi-institution and multi-cancer datasets for independent validation.

The results show that the step-wise addition of each data modality (imaging, clinical, lab, and physician’s notes) improved model performance, achieving 77% accuracy using clinical and imaging data, and 85% accuracy when laboratory and LLM-extracted symptom features were combined [152]. In a separate embedding-based implementation, CT image embeddings corresponding to the L3 level generated using the RadImageNet FM [153] were fused with embeddings from GatorTron [36] for clinical, laboratory, and free-test medical note data, enabling joint multimodal representation learning. Incorporating these radiology-derived image embeddings further enhanced cachexia prediction accuracy to 92%, underscoring the additive value of radiologic information in multimodal fusion [152]. Similarly, survival prediction improved progressively, with concordance index (C-index) gains from 0.64 using clinical data alone to 0.72 when skeletal muscle metrics, laboratory indicators, and clinical text features were added. Removing skeletal muscle metrics led to considerable declines in both cachexia diagnosis accuracy and survival prediction performance, highlighting the indispensable prognostic role of radiology-derived features [152].

From the clinical deployment standpoint, all model inferences and patient data processing, including LLM-based extraction from unstructured medical notes, are performed locally within secure computing infrastructure, avoiding reliance on external APIs and ensuring compliance with institutional data governance policies. The multimodal cachexia prediction model is intended to be portable across clinical sites because it uses routinely available inputs at diagnosis, such as CT-derived muscle biomarkers (SMA/SMI) and standard demographic/anthropometric and staging variables. Practical transfer requires consistent definition of cachexia labels, harmonization of baseline timepoints, and standardized CT series/slice selection. Inference can be performed with modest compute (CPU feasible), while model retraining benefits from GPU resources primarily for skeletal muscle segmentation. To support comparability across sites, we report performance using standardized classification metrics with confidence intervals and recommend calibration assessment and threshold selection aligned to the target site’s dataset distribution. External validation on independent multi-institution cohorts is an important next step to quantify generalizability under differing protocols and population shifts.

Together, SMAART-AI and the Multimodal AI Biomarker exemplify how imaging-derived information serves as a cornerstone for multimodal clinical AI, advancing early diagnosis, personalized intervention, and scalable cachexia management. Ongoing work is focused on expanding this framework to a multi-institutional settings through FL, supporting privacy-preserving model development across oncology centers. These efforts reflect a commitment to developing patient-centered, trustworthy AI systems designed for safe, transparent, and clinically integrated oncology practice.

### 3.3. Privacy-Preserving and Multi-Institutional AI Collaboration Using FL

We have implemented and validated a privacy-preserving FL framework to address the challenges of collaborative AI development between institutions while maintaining strict data governance standards. We implemented the NVIDIA Federated Learning Application Runtime Environment (FLARE) Software Development Kit (SDK) to enable distributed training of AI models without the need to share raw patient data. This approach is particularly well-suited for multi-institutional efforts focused on early cancer detection and the development of imaging-based biomarkers.

Initial FL simulations focused on lung cancer screening, using low-dose CT (LDCT) images from the National Lung Cancer Screening Trial (NLST) [154] and a cohort of real-world patients from our institution. This cohort varied significantly in population and characteristics of the two NLST cohorts, providing a real-world testbed to evaluate model generalization under heterogeneous conditions. To simplify onboarding across collaborating institutions, we developed a “One-Touch FL” framework in which each client site connects to the central FL server using a single Linux terminal command. This approach enables collaborators who may have limited FL experience to participate in FL runs by reducing the setup and configuration burden and mitigating the steep learning curve associated with establishing FL infrastructure. All model development orchestration, including local training, secure model updates, and aggregation, is handled centrally, reducing technical complexity at the partner sites.

Using a standard FedAvg strategy within NVIDIA FLARE, both centralized and federated models were trained on the NLST dataset to enable a direct comparison between centralized and federated training approaches. The FL global model achieved performance comparable to its centralized counterpart, demonstrating that federated training can achieve similar predictive performance to centralized model development while preserving data locality.

Next, the patient cohort from our institution was added as a third site, and a new FL model was trained across all three datasets. This model was then evaluated on a withheld test set of patients from our institution and directly compared against a centralized model trained solely on the local cohort. The task was binary cancer classification, with the model outputting either “yes” or “no”. Initially, the global FL model performed significantly worse than centralized model trained exclusively on the local dataset, underscoring a clear domain gap between the NLST data and the local cohort. To address this, the global model was fine-tuned on the local training data to better align it with the local distribution, a process known as personalized FL. These findings suggest that features learned from the NLST cohorts can be effectively leveraged in the local domain when the global model is properly adapted to site-specific data.

To evaluate NV-FLARE server–client communication and readiness for model training, we conducted cross-institutional experiments using the CIFAR-10 dataset. Training was performed between our institution and other FL sites, using a centrally hosted NV-FLARE server within a locally hosted AWS Virtual Private Cloud (VPC). This successful deployment confirmed the technical deployment of NV-FLARE for secure asynchronous federated training between geographically distributed sites. Currently, we incorporate multiple privacy preservation strategies, including secure server aggregation and optional integration of differential privacy or homomorphic encryption for enhanced protection (see Section 2.4 for discussion of additional privacy risks, attack vectors, and mitigation strategies). We are integrating several “uncertainty quantification” methods, including ensemble learning and conformal prediction, to enable model confidence estimation in distributed settings. This work is needed to enable HITL workflows in which uncertain predictions could be flagged for clinical review. Current efforts focus on scaling this the FL infrastructure to support the development of multimodal models between institutions that are part of the National Cancer Institute’s Early Detection Research Network (NCI’s EDRN) and the Privacy-Preserving Federated Learning (PPFL) consortium.

NVIDIA FLARE is an open-source software development kit designed to facilitate the implementation of FL infrastructure. The framework allows institutions to integrate existing machine learning pipelines into a federated training environment while maintaining local control of patient data. Multi-institutional FL collaborations still require appropriate data governance agreements between participating sites, which may vary depending on institutional policies. From a computational perspective, FL does not substantially increase local training requirements compared with centralized approaches. Each participating site performs standard model training, while a central aggregation server combines the model updates. The aggregation server does not require GPU resources and primarily performs parameter aggregation across sites. FL can operate with as few as two participating institutions and scale to large international collaborations. The Federated Tumor Segmentation Initiative (FeTS), for example, included 72 medical institutions across six continents, representing the largest medical deployment of federated learning to date [86]. By enabling models to learn from distributed datasets without transferring raw data, FL can particularly benefit institutions with smaller local datasets by leveraging the diversity and scale of multi-institutional data.

### 3.4. PHI/PII Redaction from Radiology Images and Reports

A critical barrier to secondary use of medical imaging data for AI model development and clinical research is the presence of PHI and Personally Identifiable Information (PII) in both DICOM metadata and pixel data. In collaboration with Impact Business Information Solutions (IBIS), we have developed a novel PHI/PII de-identification framework that addresses these challenges using a hybrid AI-driven and rule-based approach with integrated uncertainty quantification.

The framework employs a two-tiered pipeline for metadata and pixel data de-identification:

*Metadata De-Identification:* A rule-based system removes explicit PHI from DICOM headers. This is supplemented by a fine-tuned LLM-based Named Entity Recognition (NER) pipeline trained on synthetic clinical data. By simulating PHI instances using synthetic data, we ensure robust identification of sensitive fields without exposing real patient data. Additionally, fuzzy string matching techniques are applied to catch near-variants of detected PHI.

*Pixel Data De-Identification:* For image pixel data, an uncertainty-aware Faster R-CNN model detects and localizes burned-in text regions, a common but under-addressed privacy risk in medical imaging. Detected text regions undergo Optical Character Recognition (OCR) step, and the extracted text is processed through the same NER pipeline for PHI identification and removal. The model integrates uncertainty quantification, enabling the system to assess the confidence of each detection and flag uncertain cases for HITL review.

The AI-assisted hybrid workflow enhances both scalability and reliability. The use of uncertainty metrics provides transparency and allows for HITL verification when the model confidence is low. The de-identification solution has been benchmarked against regulatory standards including HIPAA, GDPR, and TCIA’s best practice guidelines. In external evaluation through the Medical Image Deidentification Benchmark Challenge (MIDI-B), across over 580,000 data elements evaluated, the system achieved over 99.8% de-identification accuracy, including successful removal of embedded PHI from diverse imaging modalities (CT, MRI, and X-ray) [155]. Quality assurance remains essential in PHI redaction workflows, as the primary concern is the occurrence of false negatives where PHI is not successfully removed. To mitigate this risk, the framework incorporates uncertainty-aware detection that flags low-confidence cases for HITL review prior to dataset release. The combination of automation and uncertainty-aware HITL review supports scalable, secure sharing of medical imaging datasets for AI development without compromising patient privacy.

A key innovation of this framework is its explicit risk calibration. Uncertain predictions are transparently quantified, enabling operational teams to set confidence thresholds, quarantine ambiguous data, and meet specific institutional or jurisdictional privacy requirements (such as “Expert Determination” under HIPAA).

The framework was designed to be institution-agnostic and deployable within local computing environments. It is currently implemented at several institutions, including Nemours, Harvard, Massachusetts General Hospital, and Emory, where it supports PHI redaction and dataset indexing workflows. Importantly, the system operates within institutional firewalls, allowing de-identification to occur without transferring data to external systems and ensuring that institutions retain full control of PHI under their existing governance frameworks. From a computational perspective, the system can operate across a range of infrastructure environments. GPU acceleration improves performance but is not required, and the pipeline can run on CPU-based systems with parallel processing to increase throughput.

## 4. Emerging Directions

### 4.1. AI-Enhanced Tumor Board Support

Medical-focused AI is gaining momentum as a decision-support tool for multidisciplinary tumor boards, where radiology provides a central foundation for oncologic care planning. By synthesizing imaging findings with pathology, genomics, and clinical records, AI has the potential to streamline case preparation, reduce administrative workload, and strengthen collaboration across specialties.

A recent study assessed 102 complex oncology cases, providing ChatGPT-4o with detailed summaries that included imaging, pathology, and prior treatment history [156]. While the model produced plausible recommendations, concordance with tumor board decisions remained modest, highlighting both the promise and the current limitations of LLM-based tumor board support. Similarly, one study tested GPT-3.5 Turbo on the data from 52 patients with non-small cell lung cancer. Each case summary included patient history, imaging reports, and guideline context. The LLM was tasked with recommending one management strategy. The agreement with the decisions of the tumor board reached 76% and the model showed a particularly high consistency in the surgical recommendations [157].

These studies demonstrate the early value of LLMs in handling clinical narratives, but they also reveal an important gap: neither incorporated radiology or pathology images together with text. So far, no published work has combined both imaging and text data in the tumor board setting. Advancing toward true multimodal integration of imaging and clinical data represents a key opportunity for medical AI. In parallel, contemporary oncology increasingly depends on molecular characterization. Radiogenomics links imaging phenotypes with underlying genomic and molecular alterations, providing insight into tumor biology beyond visual assessment alone. As next-generation sequencing becomes routine in cancer care, integrating radiologic features with genomic alterations, transcriptomic profiles, and other molecular markers within the clinical context will be essential. Future systems that can simultaneously interpret radiology, pathology, genomic, and clinical information will better capture the multidisciplinary character of tumor boards and provide more effective support for oncology decision making.

### 4.2. AI for Clinical Trial Matching

Clinical trial enrollment depends on accurately identifying patients who meet, often complex and cumbersome, eligibility rules, but this process is time-consuming is routinely done manually. AI models are now being developed to support this task by analyzing patient records along with the clinical trial enrollment criteria. A recent study showed that LLMs can effectively match patients to clinical trials by interpreting free-text eligibility criteria and comparing them with the patient’s EHR data, achieving better performance than traditional rule-based methods [158].

A promising direction for future work is to extend such approaches beyond textual records to include multimodal data sources such as diagnostic images and radiology reports. Since many eligibility criteria depend on imaging findings (e.g., tumor size, metastasis status, disease progression), VLMs that jointly reason over text and images could enable more comprehensive trial matching. Including imaging is important because reports may omit quantitative details, such as exact lesion measurements, vary by reporting style, or overlook subtle findings that are relevant to eligibility. Direct access to images allows AI systems to extract standardized biomarkers and verify reported impressions, resulting in more accurate and robust clinical trial-patient matching. Integrating these multimodal AI systems into clinical workflows would enable a major advancement in clinical trials.

### 4.3. AI-Driven Radiology Report Quality Assurance and Error Detection

Quality assurance and error detection are essential components in radiology workflows to maintain the accuracy of the radiology reports and protect patient safety. However, these tasks are both time-consuming and susceptible to human variability. LLMs are increasingly being applied to support this process by automatically detecting inconsistencies and potential errors in imaging findings. For example, a recent large-scale validation study demonstrated that GPT-4 could proofread head CT reports with high sensitivity to both factual and interpretive errors, achieving near-radiologist performance while greatly reducing review time [159].

More recently, multimodal approaches have been explored to cross-check reports directly against the underlying images [160]. A study evaluated VLMs for radiology report error detection by introducing synthetic changes, such as inserted or omitted findings, and found that models with access to chest radiographs and reports outperformed text-only systems. These findings suggest that introducing VLMs in radiology reporting workflows could move quality assurance from retrospective error detection to proactive, image-based validation that strengthens both accuracy and patient safety.

### 4.4. AI-Driven Complexity Indexing for Radiological Imaging

The U.S. radiology reimbursement system is built around CPT codes and RVUs, but these tools do not reflect the wide variation in interpretive difficulty that often exists among studies assigned the same code [161,162]. In practice, radiologists receive the same payment whether a case is straightforward or requires substantial cognitive effort [161]. Work that demands close comparison with multiple prior studies, careful reconstruction of treatment histories, or nuanced assessment of treatment response typically takes far longer and adds to the growing problem of professional fatigue and burnout [163,164]. With imaging volumes continuing to rise and staffing pressures becoming more acute, the absence of a way to represent case complexity has created a persistent mismatch between the work performed and the reimbursement assigned.

Recent advances in multimodal AI offer a way to address this gap by generating transparent, quantitative measures of interpretive complexity. These models can integrate patient comorbidities, the number and timing of prior examinations, treatment timelines, the specificity of the clinical question, and image-derived characteristics such as lesion burden, expected treatment effects, and post-operative changes. These elements can then be combined into a single complexity score that reflects the level of interpretive effort likely required. As decision-support tools become more integrated into clinical practice, a validated complexity index has the potential to give departments a clearer picture of workload, provide payers with a more appropriate basis for recognizing high-effort interpretations, and support more consistent, high-quality imaging assessment.

## 5. Conclusions

AI in radiology is steadily moving from experimental promise to practical application. Experience to date suggests that meaningful progress requires more than technical performance: local deployment, multimodal reasoning, data security, and human oversight are equally critical for building trust and ensuring clinical value. Emerging applications including multimodal AI for tumor board support, clinical trial–patient matching, automated detection of inconsistencies or errors in radiology reports, and the development of imaging complexity indices illustrate how AI can augment decision-making in ways that directly influence patient care. Future success will depend on transparent development, cross-disciplinary collaboration, and alignment with real-world radiology and oncology workflows, ensuring that AI strengthens both diagnostic quality and patient safety.

## Data Availability

No new data were created or analyzed in this study. Data sharing is not applicable to this article.

## Abbreviations

The following abbreviations are used in this manuscript:

AI	Artificial Intelligence
NLP	Natural Language Processing
CNN	Convolutional Neural Network
ViT	Visual Transformer
LLM	Large Language Model
VLM	Vision Language Model
PHI	Protected Health Information
EHR	Electronic Health Record
VQA	Visual Question Answering
FL	Federated Learning
HITL	Human-in-the-loop
